# An Approach to Fall Detection Using Statistical Distributions of Thermal Signatures Obtained by a Stand-Alone Low-Resolution IR Array Sensor Device

**DOI:** 10.3390/s25020504

**Published:** 2025-01-16

**Authors:** Nishat Tasnim Newaz, Eisuke Hanada

**Affiliations:** 1 Graduate School of Science and Engineering, Saga University, Saga 840-8502, Japan; 2 Faculty of Science and Engineering, Saga University, Saga 840-8502, Japan

**Keywords:** fall detection, low-resolution IR array, fall detection for elder people, machine learning, E-health management

## Abstract

Infrared array sensor-based fall detection and activity recognition systems have gained momentum as promising solutions for enhancing healthcare monitoring and safety in various environments. Unlike camera-based systems, which can be privacy-intrusive, IR array sensors offer a non-invasive, reliable approach for fall detection and activity recognition while preserving privacy. This work proposes a novel method to distinguish between normal motion and fall incidents by analyzing thermal patterns captured by infrared array sensors. Data were collected from two subjects who performed a range of activities of daily living, including sitting, standing, walking, and falling. Data for each state were collected over multiple trials and extended periods to ensure robustness and variability in the measurements. The collected thermal data were compared with multiple statistical distributions using Earth Mover’s Distance. Experimental results showed that normal activities exhibited low EMD values with Beta and Normal distributions, suggesting that these distributions closely matched the thermal patterns associated with regular movements. Conversely, fall events exhibited high EMD values, indicating greater variability in thermal signatures. The system was implemented using a Raspberry Pi-based stand-alone device that provides a cost-effective solution without the need for additional computational devices. This study demonstrates the effectiveness of using IR array sensors for non-invasive, real-time fall detection and activity recognition, which offer significant potential for improving healthcare monitoring and ensuring the safety of fall-prone individuals.

## 1. Introduction

The application of sensor technologies has seen tremendous progress in ensuring quality of life and safety in the areas of healthcare, assisted living, and those with mobility impairments. Among the numerous sensing modalities, infrared array sensors have proven to be an especially useful tool for detecting human activity and critical events, such as falls. Unlike traditional depth sensors or wearable devices, IR array sensors offer non-intrusive monitoring capabilities by capturing thermal patterns emitted from human bodies, making them suitable for continuous and remote monitoring in various environments [1].

The motivation behind this research was to address the difficulty in monitoring aging populations’ activity, especially the detection of falls. Falls are a major concern among older adults, often leading to severe injuries that reduce mobility and diminish quality of life. Prompt detection and intervention following a fall event can significantly mitigate these outcomes by enabling timely medical assistance and enhancing overall well-being [2]. The social impact of this research is profound, particularly in healthcare settings and home environments where older persons reside independently or with minimal assistance [3]. By deploying IR array sensor-based systems, caregivers, healthcare providers, and family members can gain real-time insights into the daily activities and well-being of persons at risk of falls. The early detection of falls not only minimizes the consequences of accidents but also reduces healthcare costs associated with prolonged hospital stays and rehabilitation.

There are several justifications for this research. The demographic is increasing worldwide due to the aging population, which calls for innovative solutions regarding health challenges attributed to aging populations, such as falls [4]. In addition, current fall detection systems mostly apply intrusive methods or wearable devices that might not be accepted well by users. Infrared array sensors offer non-contact, privacy-preserving alternatives that respect individual autonomy and allow for continuous monitoring [5]. Lastly, the strides achieved in sensor technologies, together with advanced data analytics and machine learning algorithms, have given a timely opportunity to redefine standards in fall detection and activity monitoring within the healthcare arena [6].

Falls are a leading cause of injury and mortality among the elderly population, affecting approximately 30% of adults aged 65 years and older annually [7]. According to the World Health Organization (WHO), falls result in significant morbidity and contribute to over 646,000 deaths globally each year, with adults over 60 years being the most affected [7]. In the United States, fall-related injuries generate an economic burden exceeding USD 50 billion annually [8]. Existing fall detection systems, such as those based on Kinect sensors, achieve high detection accuracies (e.g., over 90%) [9] but suffer from high costs, complex hardware setups, and privacy concerns due to video-based monitoring. Multimodal systems, while offering very high detection rates, also require significant computational resources and can compromise user privacy [10]. In contrast, our proposed system leverages non-invasive infrared array sensors, which maintain competitive detection accuracy while addressing privacy concerns. This approach ensures a practical balance between performance, affordability, and user privacy, making it particularly suitable for real-world elderly care applications.

### 1.1. Objective

The main objective of this work was to study the feasibility and performance infrared array sensor data used to classify normal static and dynamic postures based on the thermal signature, such as sitting, standing, and walking toward or away from the sensors, as well as falling down and getting up from fall events. Specifically, this work used infrared sensor data to develop strong algorithms that can perform activity recognition and fall detection in real time using infrared sensor data. Furthermore, the study attempts to verify the efficiency of the algorithms under different environmental conditions so that their reliability and usefulness in practical scenarios are guaranteed.

### 1.2. Contribution

The contribution of this research is to develop and implement a privacy-preserving system that uses infrared thermal signatures to predict fall events. The experimental analysis of this system proves that the thermal signatures of normal and falling states are statistically different. This approach will enhance safety in healthcare and assisted living environments.

### 1.3. Hypothesis

The hypothesis of this study posits that infrared thermal signatures generated during normal movements and fall events exhibit statistically distinct patterns, allowing them to be effectively differentiated through appropriate analysis.

## 2. Related Work

In [11], 3D image acquisition and fall detection were realized with two infrared sensors by which the foreground of the human body was obtained by background subtraction based on temperature differences. Localization was based on an angle of arrival algorithm with a regression-modeling-based refinement to lower the error. Fall detection was realized by simultaneously extracting features of the sensor data and optimized with k-nearest neighbor classification for high accuracy in the differentiation of fall events, guaranteeing effectiveness in real-time monitoring. The study [12] proposes a fall detection algorithm that uses MLX90640 infrared array sensors. It realizes an extended detection area and real-time ability. The subject is located before the fall detection to reduce the computational complexity. Four valuable fall features are extracted by a sliding window strategy: center of mass variation, velocity, area variation, and variance variation. Feature fusion improves the detection precision, corroborated by a support vector machine classifier. The findings of the experiment that tested the effectiveness of the system in real-life scenarios ensure better fall detection precision and feasibility. Researchers engaged in the study of the real-time systems in [9] utilized Kinect sensors that proficiently identify falls during ambulation. This method effectively eliminates false positives, such as an individual lying on the floor. By analyzing variations in the width, height, and depth of a three-dimensional bounding box, it identifies fall incidents by assessing both velocity and periods of inactivity. This methodology necessitates no prior familiarity with the scene, thus guaranteeing efficient fall detection independent of external parameter recognition. This straightforward algorithm runs fast (0.3–0.4 ms) and detects walking falls accurately while removing false positives from activities such as sitting or crouching. Instead of using traditional approaches that detect key body points, this study examines the first derivatives of a 3D bounding box to measure fall velocity with accuracy. Fall detection systems are needed to ensure the protection and well-being of the elderly and disabled people. Several approaches are now being proposed, all falling under the categories of sensor-based and machine-learning-based appropaches. Newaz and Hanada (2023) conducted an in-depth literature review on the different approaches to fall detection, elaborating on the pros and cons of each approach, along with relevant datasets, security, and privacy issues [5]. Similarly, Singh et al. (2020) studied sensor-based fall detection, bringing forth the requirement for the fusion of multiple sensors to improve accuracy [13]. The study [14] examines machine learning’s role in HAR applications using inertial, physiological, and environmental sensors, comparing ML and DL models. It highlights ML’s efficiency in low-resource cases and DL’s strength in recognizing complex activities. Abnormality prediction with machine learning and IoT have been getting popular in recent works [15]. Sixsmith et al. (2005) utilized a pyroelectric infrared sensor array for fall detection by the elderly, which provided insights into the use of thermal sensors for this purpose [16]. More recently, Han et al. (2020) used infrared ultra-wideband sensors together with convolutional neural networks to enhance fall detection accuracy [17]. The researcher of this paper [18] proposes a low-cost, device-free HAR method using an 8×8 low-resolution infrared array sensor and an LSTM-based deep learning model. The approach achieves 98.29% accuracy in recognizing activities like lying, standing, sitting, and walking, offering a high-accuracy solution for privacy-preserving smart homes. Rougier et al. (2011) used depth map video sequences to perform fall detection, one of the early examples of combining visual data with advanced processing techniques for health monitoring [19]. Kwolek and Kepski (2015) improved fall detection accuracy by fusing depth sensors with accelerometers, showing the benefits that can be gained through multimodal sensor fusion [20]. The use of direction-sensitive pyroelectric infrared sensors to improve the accuracy of fall detection systems was further explored by Liu et al. (2012) [21]. Physically challenged people can be helped by human-centered computing processes [22]. More recent developments are seen in the work of Ogawa and Naito (2020), in which fall detection based on temperature distribution extracted from the information gathered by infrared array sensors further underlines the benefits of thermal imaging in monitoring human activities [23]. Ben-Sadoun et al. (2022) conducted a systematic review of human fall detection systems based on passive infrared sensors, and the review concluded that low-resolution passive infrared sensors are adequately effective if assessed in the context of real-world applications [24]. Similarly, Nooruddin et al. (2022) conducted a review of sensor-based fall detection systems and focused on integrating different types of sensors to make the system more reliable [25]. Human health studies have been receiving attention in recent studies [26]. Newaz and Hanada (2023) presented a prototype design for human activity recognition and fall prediction based on infrared array sensors [27]. The authors of [28] demonstrated an efficient way to predict abnormal movement. Xefteris et al. (2021) performed a performance analysis in which they discussed the challenges faced during the development of multimodal fall detection systems, stating that multisensory integration can increase the accuracy of detection [10]. IoT can help to predict the normal walking pattern of humans, which can lead to fall detection [29]. Although tremendous strides have been made, there are still several challenges in the development and implementation of such systems. In addition to the fusion of multimodal data, there is great interest in several problems, including sensor accuracy and data privacy. Xefteris et al. (2021) analyze the performance challenges and limitations of their multimodal fall detection system. They give insight into the complexity of sensor fusion and real-time processing [10]. Moreover, some challenges inherent to multisensor fusion methodologies for the fall detection task are underlined by Koshmak et al. (2016), who outline the need for powerful algorithms in data processing [30]. The authors of [31] demonstrated an effective method of sensing activeness and identifying normal patterns by using a passive IR sensor. But their system cannot be used to identify specific states of movement. Another study by [32] presents a smartphone-based human activity detection system using embedded sensors like accelerometers and gyroscopes. It recognizes activities such as walking, running, and sitting, while also detecting falls using a two-stage analysis approach. Integrating IR sensors, the Internet of Things, and machine learning has dramatically improved fall detection and human activity recognition. This will continue to require research and development to overcome the current challenges and further improve accuracy, reliability, and privacy. Future efforts should focus on the development of sensor technologies and the creation of more sophisticated algorithms, as well as insuring that these systems will be integrated perfectly into everyday life.

### Novelty of the Proposed System and Comparison with Existing Systems

Table 1 presents a comparison of various sensor-based systems for fall detection and activity recognition. It includes details on sensor types, the number of sensors, their detection range, sampling frequency, accuracy, and other relevant factors such as privacy concerns, hardware complexity, and computational cost. The table also highlights the advantages and limitations of each system, making it a useful reference for evaluating different approaches to non-intrusive monitoring in healthcare contexts.

The methodology proposed in the present study utilizes IR array sensor data to distinguish between normal movement states and fall events, leveraging unique characteristics of infrared thermal imaging. This approach offers several distinctive features that are not available in existing fall detection and activity recognition systems. Table 2 compares our proposed method with existing systems that utilize IR array sensors or similar technologies for fall detection and activity recognition.

## 3. Methodology

In our previous studies [5,27,37], we analyzed previously reported fall detection methods and proposed a human activity recognition approach with IR array sensors. The system presented in this paper significantly advances our previous approach by using infrared thermal signatures in a privacy-preserving system that distinguishes between the thermal signatures of fall events and normal moving states.

### 3.1. Experiment

**Figure 1:**
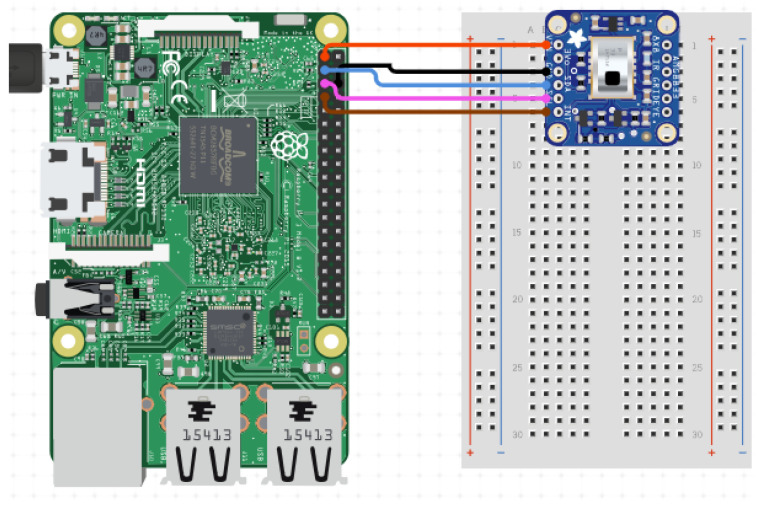
Circuit diagram of the data collection prototype.

The experimental method was carefully designed to test the ability of the IR array sensor to distinguish between normal states and fall incidents. A Raspberry Pi connected with an AMG8833 IR array sensor is used in this experiment, as shown in the circuit diagram illustrated in Figure 1.

**Figure 2:**
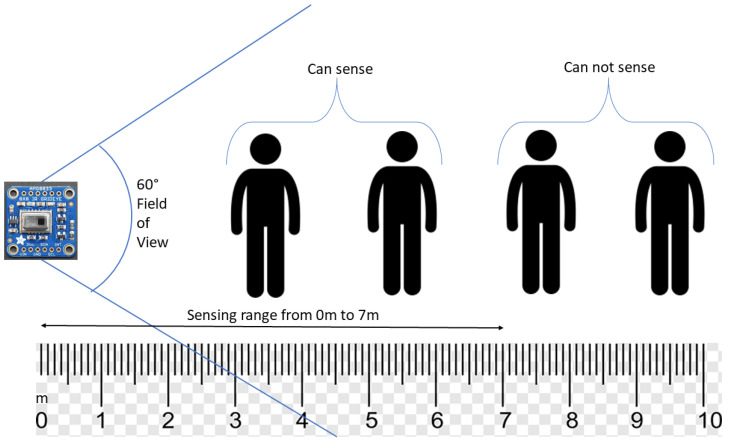
Sensing range of the AMG8833 sensor.

Key Performance Metrics of the AMG8833 IR Sensor made by Panasonic Japan are given by several important parameters: temperature range, field of view, and resolution. This sensor can accurately measure temperatures from 0 °C to 80 °C. The sensor’s field of view is approximately 60° × 60°. For optimal results, the sensor should be able to see the object of interest without any intervening obstacles, and the distance between them should not exceed seven meters. The coverage area and detection accuracy are influenced by the sensor’s height in relation to the subject’s height. Mounting the sensor too high may miss details of shorter subjects, while mounting it too low could result in it failing to fully detect taller ones. Because of the 60° × 60° field of view, the sensor’s height should be optimized to capture the entire subject within this range. Generally, placing the sensor at chest or head height ensures effective monitoring of most subjects, balancing the coverage area and accuracy while staying within its detection range. Figure 2 illustrates the sensing range of the AMG8833 sensor.

For our study, the sensor was positioned at a height of 110 cm from the ground to ensure optimal coverage of the movement area. Two human subjects participated in the experiment, each performing an average of 10 movements across different conditions. The sensor was configured to monitor a two-meter radius area in front of it, in which the subjects executed a series of predefined movements. These movements were controlled and repeated to obtain reliable data. The study involved one male and one female participant. The male participant was 166 cm tall and weighed 71 kg, while the female participant was 163 cm tall and weighed 65 kg. Both individuals were physically healthy, with no fever or any other medical conditions at the time of the study. Written consent was obtained from both participants to use their data for research purposes. The data collection took place in a controlled indoor environment with a room temperature of approximately 25 °C. For dynamic movements, participants were instructed to perform specific actions to simulate real-life scenarios. The system captures data at a sampling frequency of approximately 16 Hz, meaning it captures 16 frames per second. The data collection conditions were as follows:

- Sitting data were collected from each participant for ten minutes per experiment.
- Standing data were collected from each participant for ten minutes per experiment.
- Data for moving forward and backward were collected from each participant for ten minutes per experiment.
- Data for laying down were collected from each participant for ten minutes per experiment.
- Data for moving up and down were collected from each participant twenty times per experiment. For moving up and down, participants were asked to repeatedly squat and stand up naturally, mimicking motions such as rising from a seated position or squatting. This movement was also repeated 20 times per participant.
- Falling data were collected from each participant twenty times per experiment. They intentionally dropped from a standing position to a soft surface in a controlled environment, performing this movement 20 times.

To ensure reproducibility, the study focused on five distinct states: sitting, standing, moving forward and backward, laying down, and moving up and down. These states were chosen because they represent a range of typical activities of daily living and transitions between postures, as well as a falling state to capture critical abnormal behavior. Data were collected under controlled conditions with each participant performing the activities for a consistent duration or number of repetitions. Specifically, data for sitting, standing, moving forward and backward, and laying down were collected for ten minutes per experiment, while data for moving up and down and falling were recorded for twenty repetitions per experiment. Parameters such as consistency in execution, sensor signal stability, and clear distinction between states were used to ensure the data’s reliability. This design allowed the collection of robust datasets representative of real-life scenarios. Data were collected for 10 min for static states (sitting, standing, and laying down) to ensure sufficient data for analysis. Dynamic states (moving up/down and falling) were recorded for 20 repetitions. The choice of a 10-min duration for each data collection session was based on the need to capture a sufficient number of data for meaningful analysis. While shorter durations might also provide valuable insights, we found that 10 min offered ample time to reliably capture the thermal signatures of both normal activities and fall events. This duration allowed for the collection of diverse and representative data, accounting for variations in movements and thermal patterns that may occur during typical activities. We chose this methodology to ensure that the data was comprehensive enough to establish consistent and reliable patterns, ultimately enhancing the accuracy of the fall detection system.

The raw thermal pixel values from the AMG8833 sensor were directly used in our experiments. No additional filtering or processing was conducted. The sensor provides an 8 × 8 array of thermal data, and these raw values were used for analysis without any further conditioning. This approach was chosen to retain the full fidelity of the data captured by the sensor.

**Figure 3:**
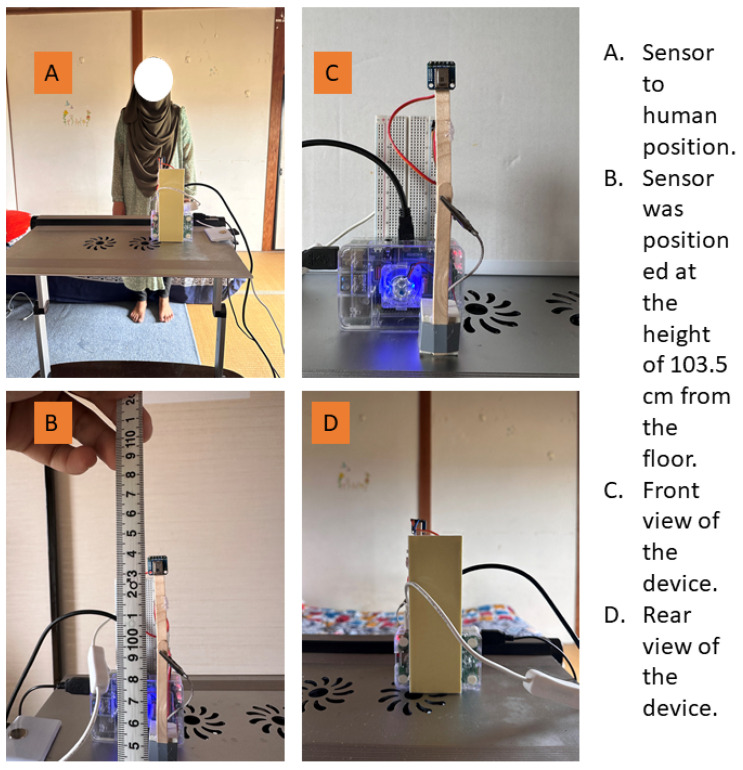
The device prototype and the experimental setup.

Data for every condition were carefully logged and processed to obtain their unique thermal patterns and transitions. The experimental setup and the controlled movement scenarios resulted in a rich dataset that was vital in proving the hypothesis that infrared array sensor data can be used effectively to distinguish between normal movements or positions and fall events. During data collection, the ambient temperature, body temperature, and clothing were not explicitly recorded. The focus of this research was to analyze the IR array data aggregated to detect fall incidents by identifying the characteristic patterns of distributions. The approach selected here emphasizes the relative distribution of heat that is captured from the IR sensors of the array, which prove to be resistant to ambient environmental conditions, such as changes in ambient temperature. This method ensures that the system performs well across different temperatures and changes in garments, making it robust and scalable for real-world applications without requiring tight control or monitoring of such parameters. The participants enrolled in the study both gave written informed consent for the use of their data. The experimental setup with the device prototype is shown in Figure 3. The system was implemented using a Raspberry Pi 4 Model B with Raspberry Pi OS. Sensor connectivity was facilitated through the wired Pi and Pi GPIO libraries. For data processing, we utilized popular libraries such as NumPy, SciPy, and Matplotlib. All source code related to this study and experiments was developed by the author.

### 3.2. Finding Data Patterns

The infrared array sensor captures infrared information by creating an 8×8 pixel array for each frame. The data of each frame were recorded and saved into a CSV file. For each of the recorded states, the sum of the pixel values in the 8×8 array was calculated for each frame, and later, histograms of the sum values were formed to depict patterns in the data. The histograms of the summed pixel values for each state were analyzed to find clear patterns. The following formula (Equation (1)) was used to compute the summation of all pixel values in a frame:

(1) $$\begin{array}{c} S_{i}=\sum_{j=1}^{64}p_{ij} \end{array}$$

In the above formula (Equation (1)), $S_{i}$ is the sum of the pixel values in the *i*-th frame. Here, $p_{ij}$ is the *j*-th pixel value in the *i*-th frame. The histogram can be used to visualize the distribution of the summed pixel values $S_{i}$. To construct the histogram, we determine the frequency $f_{k}$ of each summed value $S_{i}$ that falls into a given bin *k*.

### 3.3. Statistical Distributions and Data Comparison Methods

In this section, we provide an in-depth methodology of the statistical techniques used to compare the data from our 64-pixel infrared sensors. We focus on the Normal, Exponential, Beta, Pareto, Uniform, Laplace, Student’s t, Logistic, Gumbel, and F distributions. In addition, we use Earth Mover’s Distance to quantitatively measure how similar the empirical data distribution is to the aforementioned theoretical distributions. We preprocessed the data from the 64-pixel IR sensors to remove noise and outliers. Then, we summed up data at each pixel to construct a single dataset representing IR readings over time. For each distribution, we fit the parameters using either maximum likelihood estimation or the method of moments. The fitted parameters were then used to construct the theoretical Probability Density Function for comparison.

The Normal distribution is characterized by its mean (μ) and variance ($\sigma^{2}$). With the estimated parameters, we construct the theoretical Probability Density Function and compare it with the empirical data distribution using visual and quantitative methods.

The exponential distribution is defined by a single parameter, the rate (λ), which is the reciprocal of the mean. For our data,

(2) $$\lambda =\frac{1}{\mu }$$

This distribution is particularly useful for modeling time between events. We generate the theoretical PDF based on the estimated λ and compare it to the empirical distribution.

The Beta distribution, bounded between 0 and 1, is described by two shape parameters, α and β. The steps for our data are tp

- Normalize the data to the [0, 1] range.
- Estimate the shape parameters with methods such as maximum likelihood estimation.

The theoretical PDF is generated and compared with the normalized data.

The Pareto distribution is characterized by a scale parameter ($x_{m}$) and a shape parameter (α). For our sensor data,

(3) $$x_{m}=\min\left(x\right)$$

(4) α=MLEestimate

We then generate the theoretical PDF and compare it with the empirical data, focusing on the tail behavior.

The Uniform distribution assumes all outcomes in a specified range are equally likely. It is defined by its minimum (*a*) and maximum (*b*) values:

(5) a=min(x)

(6) b=max(x)

This distribution provides a simple baseline for comparison.

Laplace distribution, characterized by its location (μ) and scale (*b*) parameters, models data with a peak at the median and heavier tails:

(7) μ=median(x)

(8) b=MLEestimate

We generate a theoretical PDF and compare it with the empirical data.

The Student’s t distribution, useful for data with heavier tails, is defined by its degrees of freedom (ν), which is calculated with the MLE estimate. A theoretical PDF is then generated for comparison.

Logistic distribution, defined by its location (μ) and scale (*s*) parameters, has heavier tails than the Normal distribution calculated with the MLE estimate. We generate and compare the theoretical PDF with the empirical data.

Gumbel_r distribution, used for modeling extreme values, is characterized by its location (μ) and scale (β) parameters, which are calculated with the MLE estimate. A theoretical PDF is generated and compared to the data, focusing on extreme values.

The F distribution is defined by two degrees of freedom parameters ($d_{1}$ and $d_{2}$). Both are estimated from a method of moments estimate. We generate the theoretical PDF and compare it with the empirical data. It is particularly useful for variance analysis between groups.

EMD was used to quantify the similarity between the empirical data distribution and the theoretical distributions. It measures the minimum cost required to transform one distribution into another by moving distribution mass. The steps are as follows:

- Compute the empirical distribution function of the data.
- Compute the cumulative distribution function of the fitted theoretical distribution.
- Calculate the EMD as the integral of the absolute difference between the EDF and CDF.

The calculated EMD values are used to determine the best-fitting distribution for the collected data. The distribution with the lowest EMD value indicates the closest match to the empirical data. This analysis helps in understanding the underlying statistical properties of the IR sensor data and provides a basis for further statistical modeling and analysis.

Algorithm 1 provides a systematic approach to fitting various statistical distributions to the data collected from the 64-pixel IR sensors and comparing them using the Earth Mover’s Distance. The distribution with the lowest EMD value is considered the closest match to the empirical data, providing insights into the statistical properties of the sensor readings.
**Algorithm 1** Comparison of collected data with the various statistical distributions1:**Input:** Sensor data *x* from 64-pixel IR sensors2:**Output:** EMD values for each distribution3:Normalize data if required (e.g., for Beta distribution)4:**for** each distribution *D* in [Normal, Exponential, Beta, Pareto, Uniform, Laplace, Student’s t, Logistic, Gumbel_r, F] **do**5:    Fit distribution parameters $\theta_{D}$ to data *x*6:    Generate theoretical sample $y_{D}$ using fitted parameters $\theta_{D}$7:    Compute empirical distribution function of *x*8:    Compute cumulative distribution function of $y_{D}$9:    Calculate EMD between EDF of *x* and CDF of $y_{D}$10:    Store the EMD value for distribution *D*11:**end for**12:Identify distribution with the lowest EMD value as the best fit13:**Return** EMD values for all distributions

### 3.4. Insights of the Statistical Distributions

In this study, we selected ten different probability distributions to evaluate their fit to the data obtained from various human movement states, including normal states and fall events. These distributions were chosen based on their theoretical relevance, ability to model different types of data behavior, and their previous use in similar applications.

1. **Normal distribution**: A fundamental distribution often used to model continuous data that symmetrically distributes around a mean. It is useful for analyzing data with no heavy skew or outliers.
2. **Exponential Distribution**: Used for modeling time between events in a Poisson process, this distribution is suitable for data that represent waiting times or intervals between occurrences.
3. **Beta Distribution**: A flexible distribution used to model data that are confined within a specific range, ideal for normalized sensor readings that are bounded.
4. **Pareto Distribution**: A power-law distribution often used in reliability engineering and economics to model situations where a small percentage of occurrences dominate the outcomes, such as large movements or rare events.
5. **Uniform Distribution**: The simplest distribution where all outcomes have an equal probability, useful for representing cases where all outcomes are equally likely in an idealized situation.
6. **Laplace Distribution**: This distribution is useful for data with sharp peaks and heavy tails, often representing data with abrupt changes or anomalies.
7. **Student’s t Distribution**: A distribution commonly used for data that may have outliers or heavy tails. It is appropriate for real-world sensor data that might include rare extreme values.
8. **Logistic Distribution**: Similar to the Normal distribution but with heavier tails, useful for modeling logistic growth or data with more extreme variations.
9. **Gumbel Distribution (Right)**: A distribution used to model the maximum or minimum of a sample of values, appropriate for modeling extreme events such as falls.
10. **F Distribution**: Used primarily in hypothesis testing for comparing variances between groups. This distribution helps assess the variability in data across different movement types.

These ten distributions were evaluated based on their fit to the data from the sensor measurements that used the Earth Mover’s Distance metric. The goal was to identify the distribution that best represented the movement data for each state, thus enabling effective modeling of both normal and abnormal movements. The best-fit distributions for each state are presented in Section 5, and the evaluation provides valuable insights into the statistical properties of the sensor data and its potential applications for fall detection.

To build the theoretical Probability Density Functions (PDFs) for each of the distributions considered in this study, we relied on standard formulas and parameterization methods for each distribution type. The chosen distributions include Normal, Exponential, Beta, Pareto, Uniform, Laplace, Student’s t, Logistic, Gumbel, and F-distribution. These distributions were selected based on their ability to model a wide variety of patterns found in real-world data and their compatibility with the thermal signature data collected from the AMG8833 sensor. The parameters for each distribution were estimated using the data from the different movement states (normal and fall events). For example, the parameters for the Normal distribution (mean and standard deviation) were derived from the collected data corresponding to normal movements. Similarly, for distributions like Beta and Pareto, their shape and scale parameters were determined based on maximum likelihood estimation. Table 3 summarizes the key parameters and formulas used for each distribution:

In Table 3, μ and σ are the parameters for the Normal distribution, representing the mean and standard deviation, respectively. α and β are the shape parameters for the Beta distribution, while λ represents the rate for the Exponential distribution. For the Pareto distribution, the parameters α and $x_{m}$ correspond to the shape and scale respectively.

By utilizing these formulas and estimated parameters, we can generate theoretical PDFs that model the thermal signature data. These distributions are then compared to the observed data to evaluate the fit and determine the most appropriate distribution for normal and fall events.

## 4. Experiment and Data Visualization

**Figure 4:**
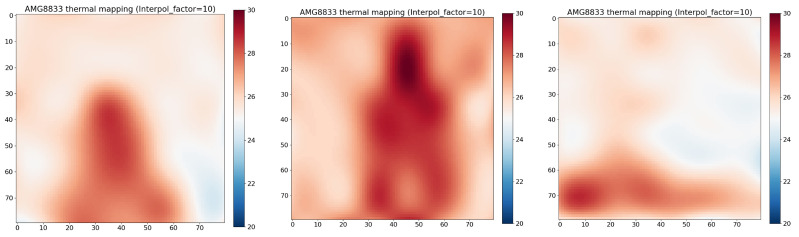
Interpolated thermal image from IR array sensor of the states: sitting (**left**), standing (**middle**), Laying (**right**).

**Figure 5:**
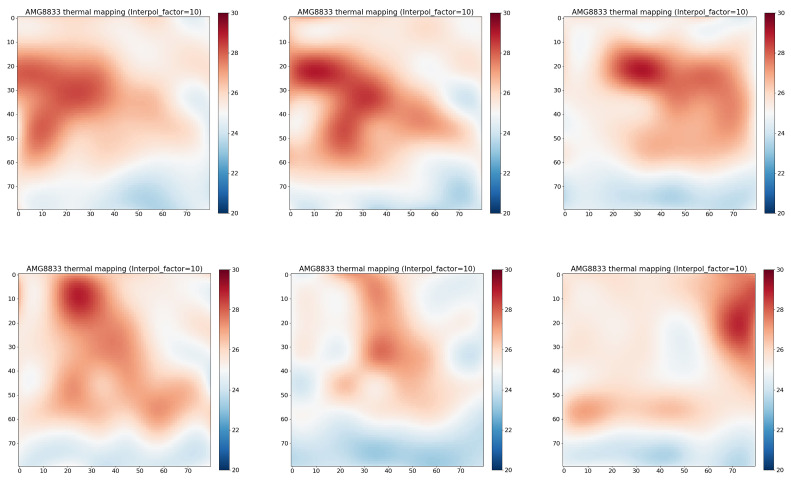
Interpolated thermal image from IR array sensor of intentionally falling onto a soft aurface.

The sensor captured infrared data and generated an 8 × 8 pixel array for each frame. The data from each frame were recorded and stored in CSV files for further analysis. For each recorded state, the sum of the pixel values in the 8 × 8 array was computed for each frame. We interpolated these low-resolution values to get a better visualization. Figure 4 illustrates an interpolated thermal image from an IR array sensor for the following states: sitting (left), standing (middle), and laying down (right). Figure 5 illustrates the interpolated thermal image from the IR array sensor for the falling state.

**Figure 6:**
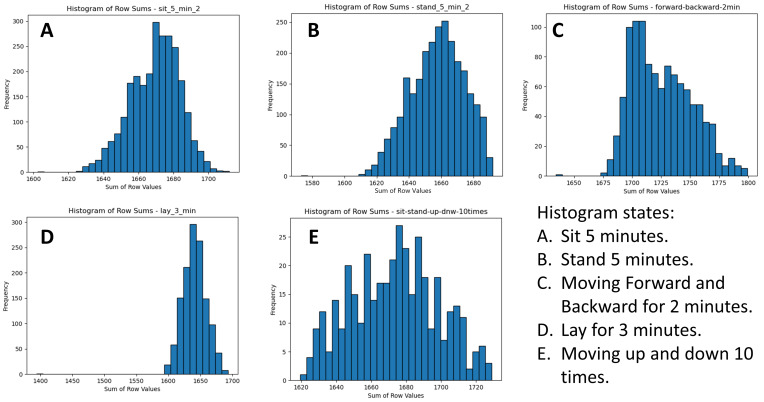
IR Array Sum Value Histograms of multiple states.

**Figure 7:**
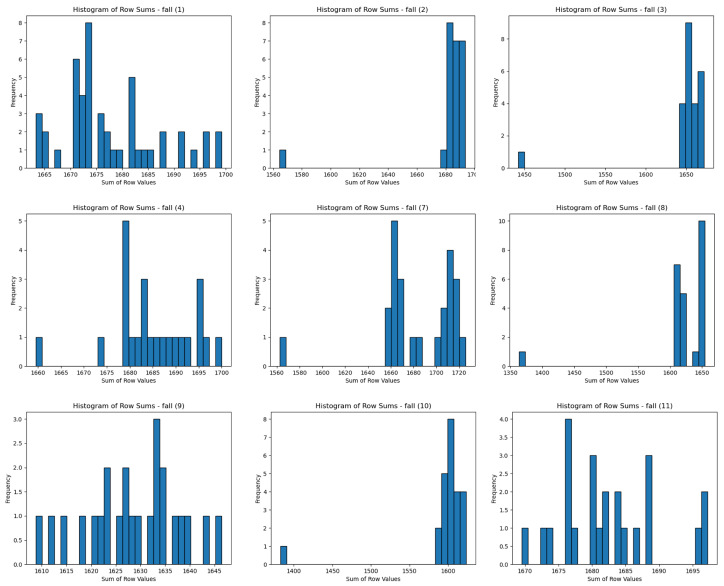
IR Array Sum Value Histograms of Multiple Falling Trials.

After this, the summed values of IR array pixels were used to plot histograms, visually representing the data distribution for each state. Figure 6 illustrates the IR array sum value histograms for various states. Figure 7 illustrates the IR array sum value histograms for multiple falling events.

## 5. Normal State and Fall Prediction Results

Comparisons of the histograms of row sum values from the various states can be performed for multiple statistical distributions. The distributions used for comparison included Normal, Exponential, Beta, Pareto, Uniform, Laplace, Student’s t, Logistic, Gumbel_r, and F. The primary goal was to identify the best-fitting distribution for each state using the EMD as a measure of similarity between the histograms and the theoretical distribution as a measure of the distance between two probability distributions. It quantifies the minimum amount of work required to transform one distribution into another, providing a meaningful comparison metric. Lower EMD values indicate a closer match between the observed data and the theoretical distribution. The results of distribution fitting for both the normal states and fall events are summarized in Table 4, which shows the EMD values and best fit distributions for normal states, and Table 5, which shows the EMD values and best fit distributions for fall states. These tables list the EMD values for each state across all considered distributions and identify the best-fitting distribution.

### 5.1. Heatmap Comparison

**Figure 8:**
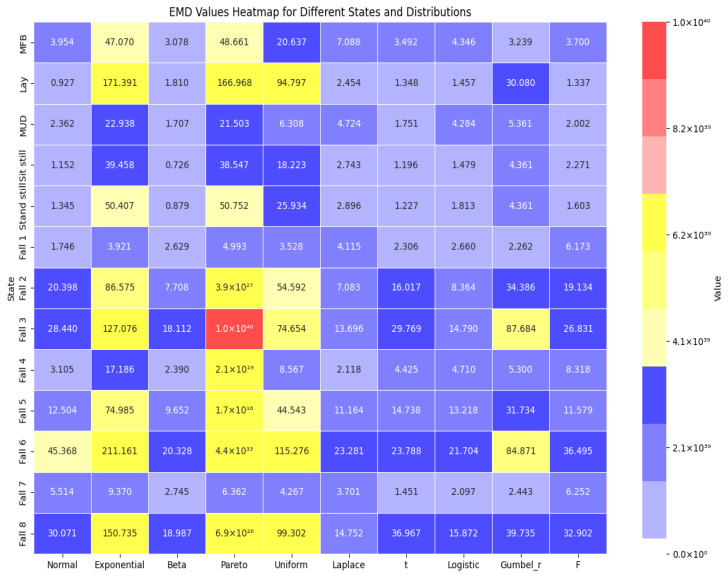
Heatmap of EMD values for different states and distributions.

The heatmap presented in Figure 8 visualizes the EMD values across various states under different probability distributions. Each cell in the heatmap corresponds to an EMD value calculated between the empirical distribution of observed data and the theoretical distribution that best fits them. The color intensity in each cell represents the magnitude of the EMD, where lighter shades denote smaller distances (indicative of better fit) and darker shades indicate larger distances (suggesting poorer fit). The color map used spans from light blue (indicating low EMD values) through yellow to deep red (indicating high EMD values). Notably, EMD values are normalized and scaled to a range from 0.0 to 1.03 × 10^40^, facilitating direct comparison across different distributions and states. This visualization highlights the variability in distribution fitting and provides insights into the efficacy of various probabilistic models in capturing the underlying data distribution characteristics across different geographic entities.

### 5.2. Normal States

**Figure 9:**
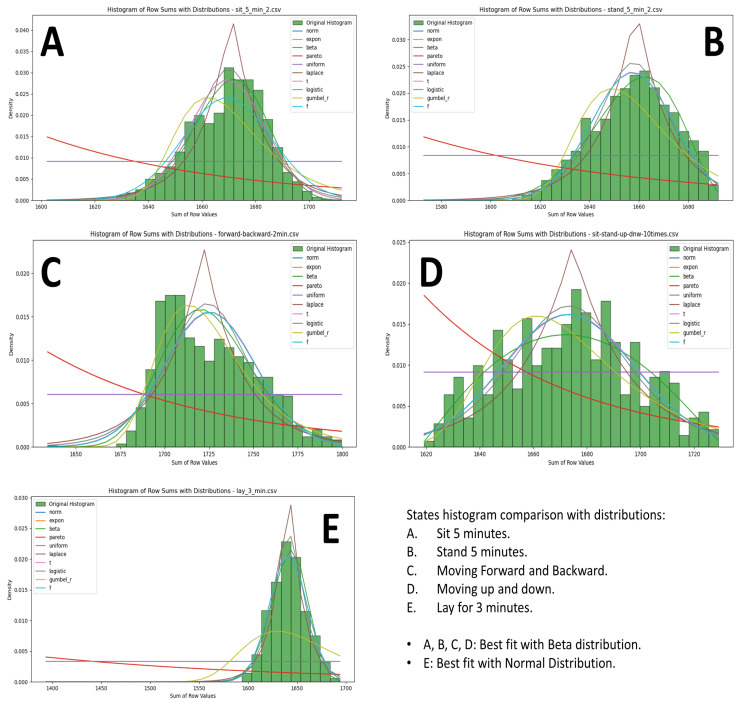
Comparison of the histograms for normal states with statistical distributions.

Table 4 shows the EMD values and best fit distributions for normal states. Figure 9 illustrates the comparison of normal state histograms with statistical distributions under the following conditions:

A: Sitting for 5 min with multiple data collection.
B: Standing for 5 min with multiple data collection.
C: Moving forward and backward with multiple data collection.
D: Moving up and down with multiple data collection.
E: Laying for 3 min with multiple data collection.

The best fit for A, B, C, and D was with the Beta distribution. The best fit for E was with the Normal distribution. The EMD values are shown in Table 4.

For normal states such as sitting, standing, moving forward and backward, moving up and down, and laying down, the histograms showed very good matches with the Beta and Normal distributions, as evidenced by the low EMD values. For instance, sitting still had an EMD value of 0.725 with the Beta distribution and 1.152 with the Normal distribution, indicating a close match. Similarly, standing still showed a low EMD value of 0.879 with the Beta distribution and 1.345 with the Normal distribution. This consistency in fitting demonstrates the regularity and predictability of typical human postures and movements.

### 5.3. Fall Events

**Figure 10:**
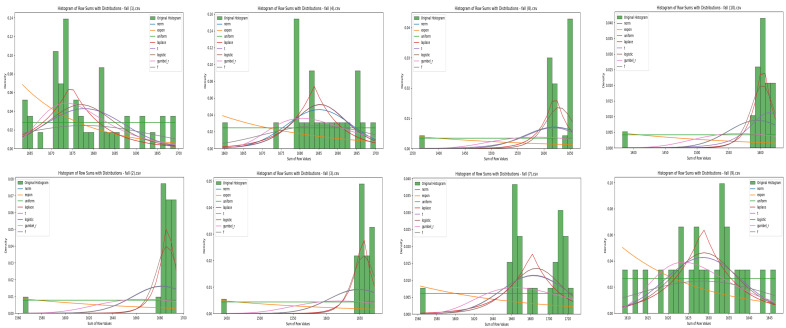
Comparison of multiple fall states’ histograms with statistical distributions.

Table 5 shows the EMD values and best fit distributions for multiple fall states. The drastic changes in EMD values between different falls, as shown in Table 5, can be attributed to the inherent variability in human movements during fall events. Each fall has unique characteristics, such as differences in speed, body orientation, impact, and recovery, which lead to distinct thermal patterns. Additionally, sensor sensitivity and environmental factors can influence the recorded data. These factors contribute to the variability observed in the EMD values, making each fall event’s thermal signature different. Figure 10 is a comparison of the histograms for multiple falls, with statistical distributions. Some fall events had a good fit with Laplace and other distributions, but they have larger EMD values. In Figure 10, we can see that no distribution lines cover most of the histograms.

The histograms of the fall events displayed increasing EMD values across most distributions, highlighting their irregular and abnormal nature. While fall events sometimes fit with the Laplace distribution, the fit was not consistent and often associated with higher EMD values. For example, Fall 2 fits best with the Laplace distribution but has a high EMD value of 7.083, indicating irregularity. Similarly, Fall 3 fits with Laplace but has an even higher EMD value of 13.695, reinforcing the abnormality of the fall movements. These higher EMD values for fall events underscore their unpredictable nature compared to normal postures.

### 5.4. Insights

The analysis illustrates the differences in data patterns between normal and abnormal states. By comparing the EMD values, it becomes evident that normal states have a consistent and predictable pattern, fitting well with the Beta distribution for sitting, standing, moving forward and backward, moving up and down and the Normal distribution for the laying down state. On the other hand, fall events show irregular and unpredictable patterns, often fitting with the Laplace distribution but with higher EMD values.

## 6. Discussion

### 6.1. Hypothesis Validation

This research hypothesized that infrared array sensor data would be useful to differentiate between normal movement states and fall events based on thermal patterns. Validating a hypothesis like this requires a huge amount of data collection and analysis under different scenarios. The data collection in this study was performed using an IR array sensor during controlled activities such as sitting, standing, forward and backward movements, up and down movements, and laying down. All activities were repeated several times to record a variety of thermal signatures for the same activity with different body postures and movements. After the data were acquired, EMD was applied to estimate the similarity between the theoretical distributions (Beta and Normal) and the measured histograms of thermal signatures. Lower values of EMD indicated that the empirical data more closely approximated the theoretical distribution and, thereby, supported the tested hypothesis that normal postural and movement states exhibit stable and predictable thermal patterns.

The results showed that EMD values for the Beta distribution were close to zero for sitting and standing activities, thus showing an extremely good fit. Similarly, in forward and backward and up and down movements, EMD values suggested a good fit with the Beta distribution. Laying down was best fitted by the Normal distribution, indicating its different thermal properties relative to the upright postures.

In contrast, fall events did not show any regular patterns. However, fall events showed high EMD values with the Laplace distribution, which indicates greater variability and unpredictability in thermal patterns when it comes to sudden falls. This confirmation supported the hypothesis that anomalous events, like falls, have thermal signatures that largely deviate from the patterns that are recorded under normal activities.

The results prove the proposed hypothesis, which will help advance safety monitoring systems based on non-intrusive infrared sensor technology in terms of better detection and response to fall incidents while preserving the privacy of the user.

Regarding the data collection duration and the number of repetitions, while we used long periods and multiple repetitions to ensure robust data, this does not imply that a subject must fall multiple times for the system to function properly. A single fall event can be detected by analyzing the thermal patterns that deviate from normal behavior. The extended data collection is intended to enhance the reliability of the system’s analysis by providing a sufficient volume of data.

### 6.2. Limitations

The proposed system has several limitations despite its innovative approach.

The IR array sensors may be sensitive to environmental influences such as temperature fluctuations or room conditions, which could impact the accuracy of thermal pattern recognition. These challenges can be mitigated by performing calibration and environmental monitoring. Additionally, fine-tuning the algorithms may improve the system’s performance in detecting intricate movements and actual fall events.

Given that the system relies on thermal mapping, it may produce deviating results when dealing with patients who have conditions such as fever, as their body temperature may resemble that seen in fall signatures. This issue can be resolved by calibrating the system for individual users, taking into account their specific thermal profiles.

Furthermore, while our study focuses on specific movement types, we acknowledge that movements such as falling may vary significantly in terms of speed, range, and other dynamic factors, which could affect detection accuracy.

The sensing field and sensitivity to height are indeed limitations of our system. The thermal sensors’ performance can be affected by the subject’s distance and height, as well as body orientation. Calibration to individual heights and body types could further help to enhance the system’s reliability.

## 7. Conclusions

This work proposes a method for the recognition of general state movements and fall event detection based on thermal signatures from a low-resolution infrared array sensor while preserving privacy. The system easily classifies common activities like sitting, standing, walking, and moving, differentiating them from fall events. The interpretation of the thermal signatures offers real-time observation and enables fast anomaly detection for better security and response to intervention in different environments. The results show the feasibility and efficiency of using infrared array sensors for non-invasive and continuous monitoring applications. This system will help to improve the current healthcare and assisted living technologies. The results we obtained included EMD values for the Beta distribution that were close to zero for sitting, standing, forward and backward, and up and down movements. Laying down was fitted by the Normal distribution, indicating its different thermal properties relative to the upright postures. In contrast, fall events did not show regular patterns. However, fall events showed high EMD values with the Laplace distribution, which indicates greater variability and unpredictability in thermal patterns when it comes to sudden falls. The achieved results showed that EMD values for the Beta distribution were close to zero for sitting (0.726), standing (0.879), forward and backward (1.707), and up and down (1.810) movements. Laying down was best fitted by the Normal distribution (0.927), indicating its different thermal properties relative to the upright postures. On the other hand, fall events did not show any regular patterns. However, fall events showed high EMD values, with the Laplace distribution achieving an average EMD of 23.281 for fall detection, indicating greater variability and unpredictability in thermal patterns during sudden falls.

Moving forward, we have identified several avenues of future work that will enhance the capabilities and applicability of the proposed system. It is possible to improve the accuracy and robustness of activity recognition and fall detection by refining the algorithmic models and calibration. A further dimension to data analytics for comprehensive insight into user activities could be gained through multi-sensor fusion approaches of IR sensors with depth sensors and accelerometers. By tackling the limitations and pursuing these research directions, the infrared array sensor-based system has tremendous potential to improve healthcare monitoring and enable better quality of life for fall-susceptible individuals.

## Figures and Tables

**Table 1:** Comparison of Various Fall Detection and Activity Recognition Systems that use different sensors.

References	[11]	[12]	[9]	[17]	[19]	[16]	[23]	[20]	[10]	Proposed System
Sensor Type	IR Sensors	MLX90640 IR Array Sensors	Kinect Sensors	IR Ultra-Wideband + CNN	Depth Cameras	Pyroelectric IR Sensor Array	IR Array Sensors	Depth Sensors + Accelerometers	Multimodal Sensors	IR Array Sensors
Sensors	2	1	1	Multiple	1	1	1	Multiple	Multiple	1
Distance to Target	5–10 m	∼7 m	∼3 m	∼10 m	∼4 m	∼5 m	∼5 m	∼4 m	Variable	∼5 m
Sampling Frequency	∼10 Hz	∼16 Hz	30 Hz	∼50 Hz	30 Hz	Low	∼16 Hz	∼50 Hz	Variable	∼16 Hz
Accuracy	High	High	High	Very High	High	Medium	High	Very High	Very High	High
Cost	Medium	Medium	High	High	High	Low	Medium	High	High	Low
Hardware Complexity	Medium	Low	High	High	High	Low	Low	High	High	Low
Computational Complexity	Medium	Medium	Low	High	Medium	Low	Medium	Medium	High	Low
Privacy	High	Medium	Medium	Low	Low	High	High	Low	Low	High
Advantages	Accurate and real-time fall detection using k-NN.	Improved detection area; real-time with SVM classification.	Fast algorithm (0.3–0.4 ms); eliminates false positives effectively.	High accuracy through CNN; effective for diverse scenarios.	Combines visual data for effective health monitoring.	Early use of thermal imaging for fall detection.	Temperature-based method; good for activity monitoring.	Multimodal fusion improves accuracy significantly.	Effective with multisensory integration for complex scenarios.	Non-invasive, privacy preservation, cost-effective, real-time.
Limitations	Limited detection area; temperature-sensitive.	Higher computational complexity for sliding window strategy.	High cost and complex hardware setup.	Complex, expensive, privacy concerns for video.	High hardware complexity; dependent on ambient lighting.	Lower accuracy; limited to simple scenarios.	Limited detection precision for dynamic activities.	High hardware and computational complexity.	High complexity and resource demands; privacy concerns.	Limited detection precision for fast dynamic activities.

**Table 2:** Novelty and comparison of the proposed system.

Key Features	Reference, Year
1-2 Utilizes IR array sensor data to differentiate between normal movement states (sitting, standing, laying down, moving forward andbackward, and up and down) and fall events based on a thermal signature or a statistical pattern while preserving privacy. The proposed system is implemented in standalone devices like Raspberry Pi, which makes it very convenient to use and of low in cost.	Proposed method
1-2 Uses IR array sensors for fall detection with machine learning, but the statistical patterns were not analyzed.	[33], 2018
1-2 Proposes a fall detection system combining IR array sensors with multi-dimensional feature fusion and SVM for enhanced accuracy, but any statistical pattern was not analyzed.	[12], 2022
1-2 Implements Kinect’s depth sensor-based fall detection system, but no statistical data patterns are formed, which requires high computing device power to implement.	[34], 2014
1-2 Uses pyroelectric IR sensor arrays for fall detection in elderly populations, focusing on passive infrared technology, but PIR sensors provide binary data that cannot be used to analyze complex patterns.	[16], 2005
1-2 Uses an IR-UWB sensor-based fall detection method using CNN algorithm for real-time monitoring, but no statistical data patterns were formed; This system requires high computing device power to implement.	[17], 2020
1-2 Uses a fall detection scheme based onIR array sensors that uses machine learning, but statistical patterns were not formed and need computer processing.	[23], 2020
1-2 Proposes deep learning classifier for fall detection based on IR distance sensor data, exploring advanced algorithms that require high computational power, and no statistical pattern was analyzed.	[35], 2022
1-2 Is a non-contact fall detection method uses MEMS infrared and radar sensors for bedside applications that may require high computational power. No statistical pattern was analyzed.	[36], 2023

**Table 3:** Theoretical Insights of the Probability Density Functions.

Distribution	Formula		Key Parameters
Normal	$f\left(x\right)=\frac{1}{\sigma \sqrt{2\pi }}e^{-\frac{{\left(x-\mu \right)}^{2}}{2\sigma^{2}}}$	(9)	μ (mean), σ (standard deviation)
Exponential	$f\left(x\right)=\lambda e^{-\lambda x}$	(10)	λ (rate parameter)
Beta	$f\left(x\right)=\frac{x^{\alpha -1}{\left(1-x\right)}^{\beta -1}}{B(\alpha ,\beta )}$	(11)	α,β (shape parameters)
Pareto	$f\left(x\right)=\frac{\alpha x_{m}^{\alpha }}{x^{\alpha +1}}$	(12)	α (shape), $x_{m}$ (scale)
Uniform	$f\left(x\right)=\frac{1}{b-a}$	(13)	a,b (lower and upper bounds)
Laplace	$f\left(x\right)=\frac{1}{2b}e^{-\frac{\left|x-\mu \right|}{b}}$	(14)	μ (location), *b* (scale)
Student’s t	$f\left(x\right)=\frac{\Gamma \left(\frac{\nu +1}{2}\right)}{\sqrt{\nu \pi }\Gamma \left(\frac{\nu }{2}\right)}{\left(1+\frac{x^{2}}{\nu }\right)}^{-\frac{\nu +1}{2}}$	(15)	ν (degrees of freedom)
Logistic	$f\left(x\right)=\frac{e^{-\frac{x-\mu }{s}}}{s{\left(1+e^{-\frac{x-\mu }{s}}\right)}^{2}}$	(16)	μ (location), *s* (scale)
Gumbel	$f\left(x\right)=\frac{1}{\beta }e^{\frac{-(x-\mu )}{\beta }}e^{-e^{-\frac{\left(x-\mu \right)}{\beta }}}$	(17)	μ,β (location and scale)
F-distribution	$f\left(x\right)=\frac{\Gamma \left(\frac{d_{1}+d_{2}}{2}\right)}{\Gamma \left(\frac{d_{1}}{2}\right)\Gamma \left(\frac{d_{2}}{2}\right)}{\left(\frac{d_{1}}{d_{2}}\right)}^{\frac{d_{1}}{2}}\frac{x^{\frac{d_{1}}{2}-1}}{{\left(1+\frac{d_{1}}{d_{2}}x\right)}^{\frac{d_{1}+d_{2}}{2}}}$	(18)	$d_{1},d_{2}$ (degrees of freedom)

**Table 4:** EMD values and best fit distributions for normal states.

Distribution	MFB	Lay	MUD	Sitting Still	Standing Still
Normal	3.954	0.927	2.362	1.152	1.345
Exponential	47.070	171.391	22.938	39.458	50.407
Beta	3.078	1.810	1.707	0.726	0.879
Pareto	48.661	166.968	21.503	38.547	50.752
Uniform	20.637	94.797	6.308	18.223	25.934
Laplace	7.088	2.454	4.724	2.743	2.896
Student’s t	3.492	1.348	1.751	1.196	1.227
Logistic	4.346	1.457	4.284	1.479	1.813
Gumbel_r	3.239	30.080	5.361	4.361	4.361
F	3.700	1.337	2.002	2.271	1.603
Best Fit	Beta	Normal	Beta	Beta	Beta

Here, MFB = Moving forward-backward, MUD = Moving sit-stand-up-down.

**Table 5:** EMD values and best fit distributions for multiple fall states.

Distribution	Fall 1	Fall 2	Fall 3	Fall 4	Fall 5	Fall 6	Fall 7	Fall 8
Normal	1.746	20.398	28.440	3.105	12.504	45.368	5.514	30.071
Exponential	3.921	86.575	127.076	17.186	74.985	211.161	9.370	150.735
Beta	2.629	7.708	18.112	2.390	9.652	20.328	2.745	18.987
Pareto	4.993	3.9 × 10^27^	1.1 × 10^40^	2.1 × 10^19^	1.7 × 10^16^	4.4 × 10^33^	6.362	6.9 × 10^26^
Uniform	3.528	54.592	74.654	8.567	44.543	115.276	4.267	99.302
Laplace	4.115	7.083	13.696	2.118	11.164	23.281	3.701	14.752
Student’s t	2.306	16.017	29.769	4.425	14.738	23.788	1.451	36.967
Logistic	2.660	8.364	14.790	4.710	13.218	21.704	2.097	15.872
Gumbel_r	2.262	34.386	87.684	5.300	31.734	84.871	2.443	39.735
F	6.173	19.134	26.831	8.318	11.579	36.495	6.252	32.902
Best Fit	Normal	Laplace	Laplace	Laplace	Beta	Beta	t	Laplace

## Data Availability

The data related to this study are available from the corresponding authors upon request.
